# Parity-Associated Differences in the Antioxidants and Fecal Microbiota of Bactrian Camels

**DOI:** 10.3390/vetsci12050440

**Published:** 2025-05-03

**Authors:** Hongxi Du, Jianxiong Xu, Hongcai Zhang, Jianjun Li, Fei Wang, Huan Li, Sarula Han, Jiri Gala, Jilite Wang

**Affiliations:** 1 Innovative Team for Hetao Agricultural Products’ Nutrition and High-Value Utilization, Department of Agriculture, Hetao College, Bayannur 015000, China; hongxidu@aliyun.com (H.D.); jrgl970715@126.com (J.G.); 2 School of Agriculture and Biology, Shanghai Jiao Tong University, Shanghai 200042, China; jxxu1962@sjtu.edu.cn (J.X.); hczhang@sjtu.edu.cn (H.Z.); 3 Inner Mongolia Yinggesu Biotechnology Co., Ltd., Bayannur 015000, China; m13314784888_1@163.com (J.L.); 18047826370@163.com (F.W.); 15248868522@163.com (H.L.); 4 Hulunbuir Agricultural and Animal Husbandry Institute, Hulunbuir 021008, China; hansarula2022@163.com

**Keywords:** camel, milk, serum, antioxidants, feces, microbiome, total antioxidant ability, non-oxidative pentose phosphate pathway, ANAGLYCOLYSIS-PWY

## Abstract

Camels survive in deserts through unique, adapted metabolic and immunological processes that are normally lethal to other species. Antioxidants and the gastrointestinal microbiota play major roles in redox homeostasis, yet they remain unexplored in camels to date. The objectives of this study were to characterize the dynamics of milk antioxidants, serum antioxidants, and the fecal microbiome of lactating Bactrian camels with different parities. In total, 30 lactating camels were selected and categorized into the following 3 groups: 10 were assigned to the first parity group (P_1), 10 were classified into the third parity group (P_3), and 10 belonged to the fifth parity group (P_5). The antioxidant parameters of the lactating camels were determined in milk and serum. The fecal microbial community of lactating camels was assessed using 16S rRNA amplicon sequencing, and the resulting library was sequenced on an Illumina NovaSeq platform. The amount of total antioxidant capacity (T-AOC) and antioxidant activity for polypepetides (DPPH) in the third parity was the highest among the groups both in the camel milk and its serum. In the case of hydroxyl radical (OH) and SOD, the amounts were 9.62 U/mL (SEM = 0.4950) and 13.64 U/mL (SEM = 0.5144), respectively, for P_1 in the serum, which were extremely significantly greater than those of the other groups. The Shannon index was significantly different between the P_1 group and either the P_3 or P_5 group. Additionally, Simpson’s diversity index significantly differed between the P_1 group and the P_5 group. Furthermore, the number of OH in camels is positively associated with the metabolic pathway of non-oxidative pentose phosphate pathway and ANAGLYCOLYSIS-PWY. In conclusion, this study revealed that different parities were associated with distinct levels of antioxidant parameters and fecal microbial ecologies in lactating Bactrian camels, where parity affects metabolic and microbial health. Understanding these dynamics in camels could optimize their nutritional management, enhancing their welfare in challenging environments.

## Simple Summary

In certain nations, there has been an increase in the breeding and management of dromedary and Bactrian camels for milk production. Parity is an important physiological process, and this study was designed to reveal the dynamics of antioxidant parameters and the fecal microbiome. Antioxidant parameters affect animal health and welfare, and the gastrointestinal microbiome can be influenced by environmental changes that the host may face. This study focused on the possible relationships among parity, antioxidants, and the gastrointestinal microbiome. This study revealed changes in antioxidants in milk and serum and in the fecal microbial communities of lactating camels of different parities. Camel behavior and welfare are new phenomena in the area of animal production, so this study may provide scientific information for the friendly management of lactating camels.

## 1. Introduction

In deserts, camels hold irreplaceable significance for rangeland ecology [1] and food production [2]. Camels have evolved unique traits and physiological mechanisms to thrive in arid climates, with dromedaries and Bactrian camels adapting to these environments [3,4]. Camels survive in arid climates through unique, adapted metabolic and immunological processes that are normally lethal to other species [5]. However, antioxidants play a major role in redox homeostasis in animals by controlling the overproduction of prooxidants [6]. A better understanding of redox changes may provide a deeper understanding of the pathophysiology and help in the development of control strategies for healthy animals related to growth, pregnancy, parturition, and lactation periods [7].

In camels, research on antioxidants and oxidative stress has focused mainly on the peripartum, pregnancy, and early lactation periods. Research on the effects of reproductive disorders on milk production and proinflammatory cytokines has revealed that the use of prooxidants may be necessary for early diagnosis to prevent related diseases in camels suffering from reproductive disorders [8]. Furthermore, periparturient camels experience substantial oxidative stress, particularly at parturition and the week after calving, as indicated by increased total antioxidant capacity [9]. Moreover, studies have revealed linkages between steroid hormones and antioxidant parameters during pregnancy and early lactation in female camels [10]. Dynamic changes in serum or milk antioxidant parameters as a result of lactation in the postpartum period in camels have also been reported [4].

The gut microbiome and its metabolites have significant effects on host health, immunity, metabolism, and neurobehavioral traits in mammals [11,12]. Conversely, environmental factors such as diet, host genetics, parity, sex, and animal health influence the composition of the microbial community both quantitatively and qualitatively [13,14]. Camels provide the necessary physiological conditions for microbial growth, as they rely on these microbes for the digestion of shrubs and nutrient supply [15]. However, camels possess a distinct gastrointestinal system consisting of the rumen, reticulum, and abomasum but lacking the omasum, which suggests that their gastrointestinal microbiome is likely distinct [16].

The number of times a woman has given birth has long-term implications for women’s physical and mental health [17,18]. In humans, the vaginal microbiota composition is strongly associated with advancing gestational age and parity [19]. In dairy cattle, increasing parity is inversely associated with survival and reproduction [20]. The relative abundance of Actinobacteria was greater in primiparous cows than in multiparous cows, emphasizing the association of the uterine microbiota with parity [21]. In multiparous Holstein dairy cattle, reactive oxygen species and malondialdehyde increase oxidative stress during the early lactation period [22]. High-yield dairy cattle commonly exhibit high levels of oxidative parameters and are exposed to inflammatory stimuli during parturition and early lactation [23,24]. Cows in the first and second lactations had greater levels of antioxidants than cows in their third and greater lactations did [25].

In camels, milk proteins and free fatty acids are markedly affected by parity in traditional rangeland production systems [26]. The milk fat of primiparous camels, an intensive production system, appears to have relatively high nutritional value, as it contains relatively high levels of unsaturated fatty acids [27].

However, the dynamics of antioxidant parameters and gastrointestinal microbes remain largely unexploited for camels with different parities. Therefore, this study aims to characterize the differences among antioxidants in milk, antioxidants in serum, and the fecal microbial community in lactating Bactrian camels with different parities.

## 2. Materials and Methods

This study was carried out in accordance with the recommendations of the Instructive Notions with Respect to Caring for Experimental Animals of the Ministry of Technology of China. The protocol was approved by the Ethics Committee of Hetao College (protocol code HTDX2330 and date of approval 7 July 2023).

### 2.1. Experimental Design, Animals, and Sample Collection

Prior to the first parturition, the camels remained in the traditional rangeland production system, where the main vegetation belongs to Amaranthaceae, Fabaceae, Zygophyllaceae, Poaceae, and Liliaceae. They are capable of ingesting shrubs, such as *Caragana tibetica*, *Haloxylon ammodendron*, *Caragana korshinskii*, and *Sarcozygium xanthoxylon*. After parturition and 60 days of lactation, the lactating camels were sent to the intensive production system, which was housed in an open yard that provided total mixed rations (TMR), and the diet composition and nutrient levels are displayed in Table 1. The camels of different parities were housed together in the same open yard, and the TMR was provided three times a day for the camels ad libitum.

The animals were group-fed a diet of 20 kg/head/day, and drinking water and alkali stones were provided all day. After the lactating period, the female camels returned to the traditional rangeland until the next parturition. The lactating Bactrian camels, with a mean body weight of 473 kg (ranging from 460–490 kg), and their kids, with a mean body weight of 154 kg (ranging from 140–160 kg), included in this research, were obtained from Inner Mongolia Yinggesu Biotechnology Co. Ltd. (Urad-North Banner, Bayannur, China).

There were 642 lactating camels in total, the majority of the lactating camels’ parities ranged from 1st to 5th parity, and the parturition time ranged from mid-March to mid-April. In total, 30 camels with parturition times ranging from 20 March to 25 March were selected, and the lactating camels were categorized into the following 3 groups: P_1 (10 female lactating camels, 1st parity), P_3 (10 female lactating camels, 3rd parity), and P_5 (10 female lactating camels, 5th parity). The mean ages for the P_1 group was 5 years (average milk production of 1.53 kg/d per camel), 9 years for the P_3 group (average milk production of 2.62 kg/d per camel), and 14 years for the P_5 group (average milk production of 2.99 kg/d per camel).

The lactating day ranged from the 155th day to the 160th day, and sampling was conducted on 30 August. Blood samples from the respective camels were collected from the jugular vein and placed into 10 mL plain vacutainer tubes after the morning milking, and 15 mL of blood was collected from each individual camel. The serum was separated immediately and stored at −20 °C. The respective individual milk and fecal samples were obtained from lactating camels by collecting the samples from each camel into sterile frozen tubes (Falcon^TM^ 10 mL conical) and collecting 10 mL of milk and feces, respectively. The collected milk and fecal samples were then stored at temperatures of −20 °C and −80 °C, respectively. Finally, antioxidant analysis of milk and serum was conducted within twenty days, while microbial DNA extraction and 16S rRNA sequencing were performed within thirty days.

### 2.2. Antioxidant Analysis in Milk and Serum

The total antioxidant capacity (T-AOC), catalase (CAT), antioxidant activity for hydroxyl radical (OH), superoxide dismutase (SOD), and glutathione peroxidase (GSH), and antioxidant activity for polypeptides (DPPH) in milk and serum were examined via commercial kits (Nanjing Jiancheng Biotech Institute, Nanjing, China).

### 2.3. Microbial DNA Extraction and 16S rRNA Sequencing

The total genomic DNA from the samples was extracted using the hexadecyltrimethylammonium bromide method. The concentration and purity of the DNA were assessed via 1% agarose gels and a Nanodrop spectrophotometer (Thermo Fisher Scientific, Madison, WI, USA). The DNA was diluted to a concentration of 1 ng/µL in sterile water. The 16S rRNA genes, specifically the V3~V4 regions, were amplified via the specific primers 341F (5′-CCTAYGGGRBGCASCAG-3′) and 806R (5′-GGACTACNNGGGTATCTAAT-3′) with barcodes, and the barcode sequence was dual-indexed for each sample. All PCRs were performed with 15 µL of Phusion^®^ High-Fidelity PCR Master Mix (New England Biolabs, Beijing, China), 2 µM forward and reverse primers, and about 10 ng of template DNA. The thermal cycling protocol included an initial denaturation step at 98 °C for 1 min, followed by 30 cycles of denaturation at 98 °C for 10 s, annealing at 50 °C for 30 s, and elongation at 72 °C for 30 s, with a final extension step at 72 °C for 5 min. The PCR products were then mixed with an equal volume of buffer and subjected to electrophoresis on a 2% agarose gel for detection. The PCR products at equal ratios were pooled together, and the mixture was purified via universal DNA purification kits (TianGen, Tianjin, China). The PCR was performed in three technical replicates for each individual sample. The technical replicate (3 independent DNA extractions/sequencing per set) were designed to verify the experimental robustness.

### 2.4. Libraries Generated, Illumina NovaSeq Sequencing, and Bioinformatics

The NEB Next^®^ Ultra DNA Library Prep Kit (Illumina, San Diego, CA, USA) was utilized to generate sequencing libraries following the manufacturer’s guidelines. Index codes were added to the libraries. The quality of the libraries was evaluated via an Agilent 5400 system (Agilent Technologies Co., Ltd., Santa Clara, CA, USA). Finally, the libraries were sequenced on an Illumina NovaSeq platform (Illumina, San Diego, CA, USA), resulting in the generation of 250 bp paired-end reads.

The analysis was performed following the “Atacama soil microbiome tutorial” provided in the Quantitative Insights into Microbial Ecology (QIIME2) documentation, along with customized program scripts (https://docs.qiime2.org/2019.1/; accessed on 10 February 2023). Normalization was conducted on the read counts before downstream analysis using the Shapiro-Wilk test. The total good depth was 99.51%, and the mean read count was 79,161 for the samples. In summary, the raw data FASTQ files were imported into a format compatible with the QIIME2 system via the QIIME tools import program. The demultiplexed sequences from each sample were subjected to quality filtering, trimming, denoising, and merging. The QIIME2 dada2 plugin was utilized to identify and eliminate chimeric sequences, resulting in the generation of an amplicon sequence variant (ASV) feature table [28]. To assign taxonomic classifications, the QIIME2 feature-classifier plugin was employed to align the ASVs to a pretrained GREENGENES 13_8 99% database (trimmed to the V3-V4 region defined by the 338F/806R primer pair), producing a taxonomy table [29]. The QIIME2 feature-table plugin was used to remove any mitochondrial or chloroplast sequences that could contaminate the analysis. Unless otherwise specified, default parameters were employed for the analysis. Additionally, the potential functional profiles of the microbial communities, including Kyoto Encyclopedia of Genes and Genomics (KEGG) pathways and Enzyme Commission (EC) annotations, were predicted via phylogenetic investigation of communities by reconstruction of unobserved states (PICRUSt) [30]. The pathway abundances were normalized.

Diversity metrics were calculated using the core-diversity plugin in QIIME2. Alpha diversity indices at the feature level, including observed operational taxonomic units (OTUs), the Shannon diversity index, and Faith’s phylogenetic diversity index, were computed to assess the microbial diversity within each sample. The rarefaction cutoff level was 38084. The Wilcoxon test was employed to evaluate the significance of differences between the groups. To assess the structural variation in microbial communities across samples, beta diversity distance measurements, specifically weighted UniFrac, were performed. Principal coordinate analysis (PCoA) of the fecal microbial communities was performed using the FactoMineR package and visualized via the ggplot2 package in R software (version 3.5.2). The relative abundances of the microbes, KEGG, and ECs were ranked by QIIME and visualized via the ggplot2 package in R.

### 2.5. Statistical Analysis

The statistical analysis accounted for multiple testing corrections to avoid false positives. To identify taxa with differing abundances among groups, linear discriminant analysis effect size (LEfSe) was employed, and significant differences were defined as linear discriminant analysis (LDA > 4) and *p* < 0.05. Additionally, principal component analysis (PCA) of all the samples was performed using the FactoMineR package and visualized using the ggplot2 package in R.

One-way ANOVA of the amounts of T-AOC, OH, SOD, GSH, and DPPH in milk and serum was performed by SAS (v9.4). Duncan’s test was carried out to determine significant differences at *p* < 0.05.

Considering the false discovery rate, Spearman’s rank correlations were calculated to explore the relationships among the top 20 genera, top 20 MetaCyc, top 20 KEGG, and top 20 ECs with the oxidative stress parameters, and these correlations were visualized via the pheatmap package in R. There were significant correlations (*p* < 0.05), highly significant correlations (*p* < 0.01), and extremely significant correlations (*p* < 0.001).

## 3. Results

### 3.1. Dynamics of Antioxidants in the Lactating Camel

As shown in Table 2, concerning the P_3 group, the amounts of T-AOC (86.09 mg/mL), SOD (68.17 U/mL), and DPPH (205.43 μg Trolox/mL) were significantly greater than those in the other groups. In the case of OH, the amounts were 16.75 U/mL and 16.08 U/mL for the P_3 and P_5 groups, respectively, which were significantly greater than those of P_1. Concerning the P_1 group, the amount of CAT (2.87 U/mL) was greater than that in the other groups.

As shown in Table 3, concerning the P_3 group, the amounts of T-AOC (5.30 mg/mL) and DPPH (126.04 μg Trolox/mL) were significantly greater than those in the other groups. In the case of OH and SOD, the amounts were 9.62 U/mL and 13.64 U/mL for P_1, respectively, which were significantly greater than those of the other groups. In the P_5 group, the amount of SOD (11.50 U/mL) was greater than that in the P_3 group.

### 3.2. Dynamics of the Fecal Microbial Community in the Lactating Camel

All the sequences were deposited in the NCBI Sequence Read Archive (SRA) at the accession number PRJNA964482. A total of 2,374,823 reads were obtained through the sequencing of bacterial and archaeal 16S rRNA genes. Of these, 99.42% were assigned to bacteria, and 0.58% were assigned to archaea. In total, 8389 OTUs were identified, with 32 phyla, 98 classes, 148 orders, 187 families, 200 genera, and 59 species.

**Figure 1:**
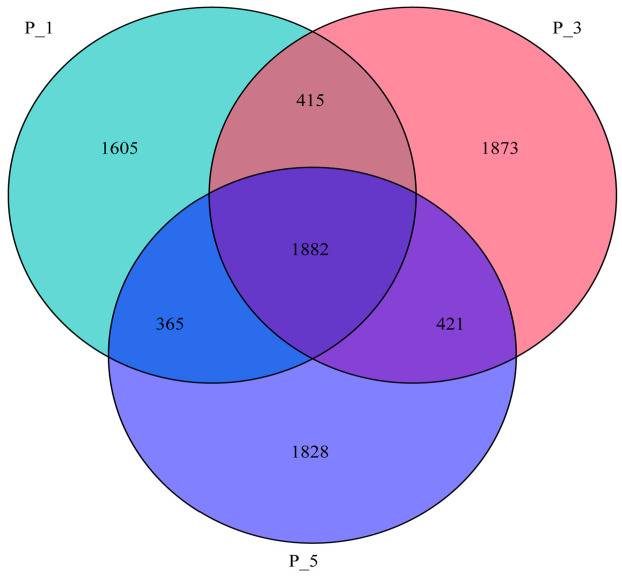
Venn diagram showing the overlap of OTUs across the groups of the fecal microbial community in lactating Bactrian camels. Green represents P_1 (10 female lactating camels, 1st parity), red represents P_3 (10 female lactating camels, 3rd parity), and blue represents P_5 (10 female lactating camels, 5th parity).

A Venn diagram (Figure 1) revealed that there were 1882 common observed operational taxonomic units (OTUs) among the three groups. Additionally, 1605, 1873, and 1828 OTUs were observed in groups P_1, P_3, and P_5, respectively.

**Figure 2:**
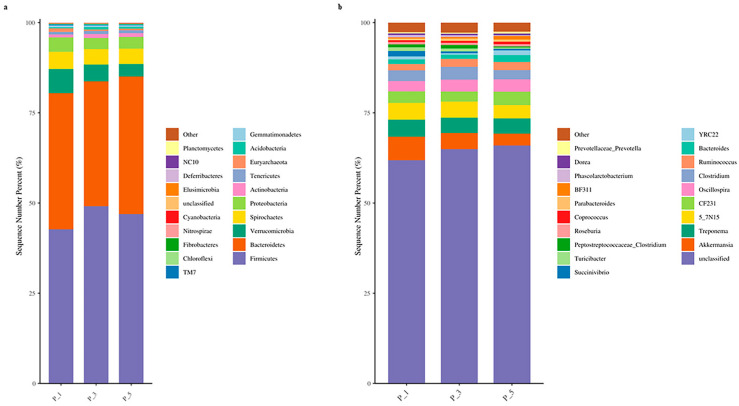
Bar graphs showing the top 20 relatively abundant phyla (**a**) and genera (**b**) in lactating Bactrian camel feces. The horizontal axis represents the different pairs of lactating camels: P_1 (10 female lactating camels, 1st parity), P_3 (10 female lactating camels, 3rd parity), and P_5 (10 female lactating camels, 5th parity). The vertical axis illustrates the proportion of sequences attributed to the relevant phylum (**a**) or genus (**b**). The colors of the bar graphs from top to bottom align with the colors for each taxon on the right, ranging from the lowest to the greatest relative abundances, with the exception of ‘Other’. At the phylum level, sequences that could not be assigned were categorized as ‘unclassified’, whereas ‘Other’ denotes the cumulative percentage of all other phyla that did not make the top 20. Similarly, at the genus level, ‘Other’ represents the cumulative percentage of all other genera that fell outside the top 20.

Figure 2a illustrates the top 20 most abundant phyla. Among them, Firmicutes (46.25%) was the most prevalent, followed by Bacteroidetes (36.84%), Verrucomicrobia (4.92%), Spirochaetes (4.44%), Proteobacteria (3.44%), and Actinobacteria (0.97%). Figure 2b presents the top 20 most abundant genera. In addition to the unclassified genera (64.24%) and other genera (2.64%), the most abundant genus was *Akkermansia* (Verrucomicrobia), with a relative abundance of 4.77%, followed by *Treponema* (Spirochaetes), with a relative abundance of 4.38%. Within the Bacteroidetes phylum, *5_7N15* accounted for 4.26% of the relative abundance, *CF231* accounted for 3.25%, and *Bacteroides* accounted for 1.48%. In the Firmicutes phylum, *Oscillospira* accounted for 3.19% of the relative abundance, *Clostridium* accounted for 3.02%, and *Ruminococcus* accounted for 2.04%.

**Figure 3:**
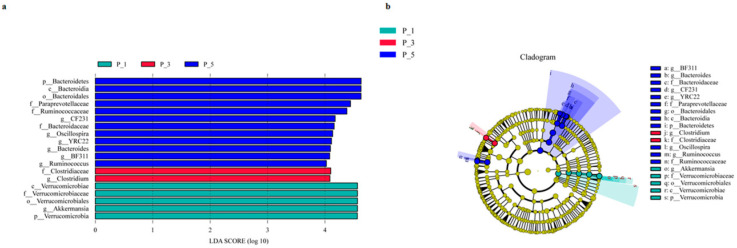
LEfSe analysis of the fecal microbiome (**a**) and cladogram (**b**) in lactating Bactrian camels. The horizontal axis represents the different pairs of lactating camels: green, red, and blue correspond to the P_1, P_3, and P_5 groups, respectively. (**a**) Each horizontal bar represents a specific taxon, with the length of the bar indicating the linear discriminant analysis (LDA) score. (**b**) Fan-shaped areas of the same color label the related taxon, with each circle ligature representing a distinct taxon. Yellow signifies no significant difference among the groups, whereas other colors denote the biomarkers for the respective group.

In this study, the notation p__ represents phylum, c__ denotes class, o__ stands for order, f__ signifies family, and g__ indicates genus. LEfSe stands for linear discriminant analysis, and LDA stands for latent Dirichlet allocation. LEfSe analysis (LDA > 4) at the genus level, as shown in Figure 3a and the cladogram shown in Figure 3b, revealed that f_Clostridiaceae (LDA = 4.1010, *p* = 0.0201) and g_*Clostridium* (LDA = 4.0865, *p* = 0.0201) were significantly more abundant in the P_3 group. *Clostridium* species are often linked to butyrate production, reflecting niche-specific functions in this group.

In the P_1 group, p__Verrucomicrobia (LDA = 4.5658, *p* = 0.0081), c__Verrucomicrobiae (LDA = 4.5658, *p* = 0.0093), o__Verrucomicrobiales (LDA = 4.5658, *p* = 0.0093), f__Verrucomicrobiaceae (LDA = 4.5658, *p* = 0.0093), and g__*Akkermansia* (LDA = 4.5658, *p* = 0.0093) were more abundant than in the other groups. *Akkermansia* is strongly associated with gut barrier integrity and metabolic health. The dominance here suggests that lactating camels with 1st parity may present enhanced mucosal protection.

In the P_5 group, o__Bacteroidales (LDA = 4.6282, *p* = 0.0008), f__Bacteroidaceae (LDA = 4.0829, *p* = 0.0003), and its two genera, g__*BF311* (LDA = 4.0829, *p* = 0.0003) and g_*Bacteroides* (LDA = 4.0945, *p* = 0.0006), as well as f__Paraprevotellaceae (LDA = 4.4450, *p* = 0.0065) and its two genera, g__*CF231* (LDA = 4.1776, *p* = 0.0231) and g__*YRC22* (LDA = 4.1146, *p* = 0.0060), were significantly more abundant than those in the other groups. *Bacteroides* and Ruminococcaceae are key degraders of dietary fiber, producing short-chain fatty acids (SCFAs). Furthermore, f__Ruminococcaceae (LDA = 4.3818, *p* = 0.0013) and its two genera, g__*Ruminococcus* (LDA = 4.0251, *p* = 0.0159) and g__*Oscillospira* (LDA = 4.1349, *p* = 0.0019), were significantly more abundant in the P_5 group than in the other groups. *Oscillospira* is often linked to lean phenotypes, suggesting potential metabolic benefits in lactating camels with a 5th parity.

As presented in Table 4, there were no significant differences in Faith’s phylogenetic diversity (faith_pd) or OTUs among the P_1, P_3, and P_5 groups. Unlike the Shannon index, faith_pd accounts for the evolutionary history of taxa, for which a higher value indicates greater phylogenetic diversity.

However, the Shannon_entropy index was significantly different between the P_1 group and either the P_3 or P_5 group (*p* = 0.015). Additionally, Simpson’s diversity index was significantly different between the P_1 group and the P_5 group (*p* = 0.023).

**Figure 4:**
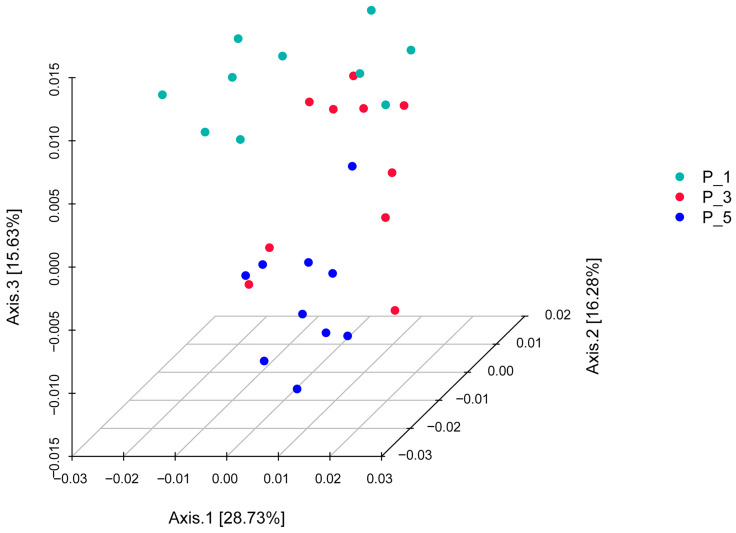
Principle coordinate analysis (PCoA) 3D analysis of the fecal microbial communities associated with different parities in lactating Bactrian camels. In the color scheme, green denotes P_1 (10 female lactating camels, 1st parity), red signifies P_3 (10 female lactating camels, 3rd parity), and blue represents P_5 (10 female lactating camels, 5th parity). Each plot corresponds to a single sample, and plots of the same color belong to the same group.

As shown in Figure 4, 3D principal coordinate analysis (PCoA) based on weighted UniFrac revealed that the overall microbial communities of the P_1, P_3, and P_5 groups were clustered together. PCoA axis 1 accounted for 28.73% of the variation, PCoA axis 2 accounted for 16.28% of the variation, and PCoA axis 3 accounted for 15.63% of the variation.PCoA Axis description confusing: revise “”.

### 3.3. Dynamics of MetaCyc Pathways in Lactating Camel Feces

**Figure 5:**
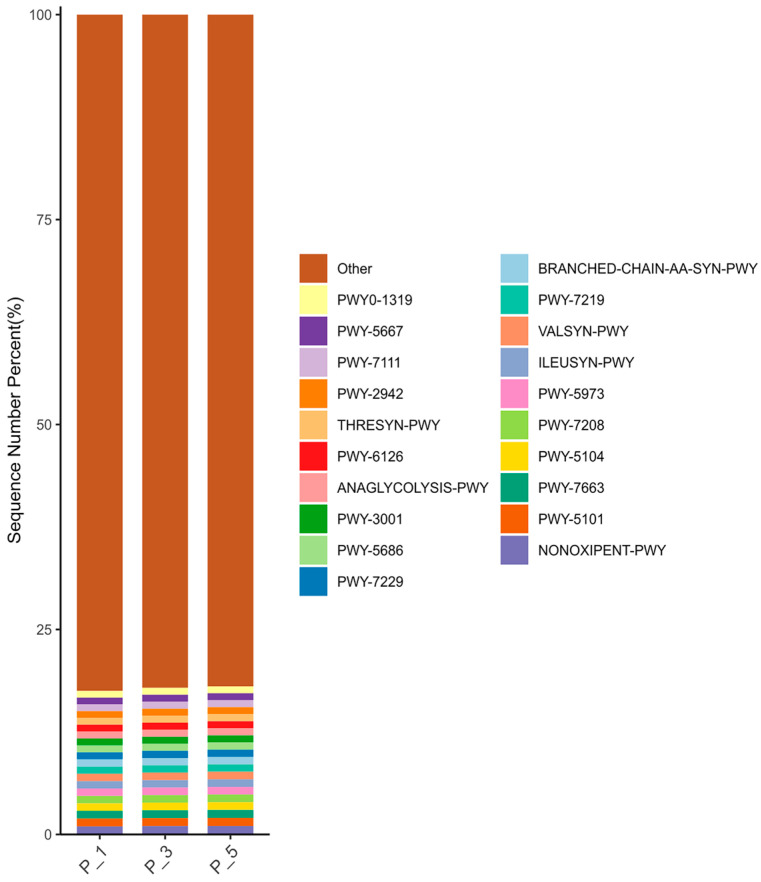
Bar graphs describing MetaCyc pathways in lactating Bactrian camel feces. The horizontal axis represents parities P_1 (10 female lactating camels, 1st parity), P_3 (10 female lactating camels, 3rd parity), and P_5 (10 female lactating camels, 5th parity). The vertical axis, or Sequence Number Percent, visualizes the percentage of sequences attributed to the corresponding MetaCyc pathways. The color scheme for the bar graphs, moving from top to bottom, corresponds to the colors for each MetaCyc pathway displayed on the right, ranging from the least to the most relative abundances, with ‘Other’ being an exception that did not make the top 20.

As depicted in Figure 5, NONOXIPENT-PWY had the highest relative abundance of metabolic pathways, accounting for 1.01% of the total. The MetaCyc pathways with relative abundances greater than 0.9% were PWY-5101, PWY-7663, PWY-5104, PWY-7208, PWY-5973, ILEUSYN-PWY, VALSYN-PWY, and PWY-7219. The relative abundances of MetaCyc pathways greater than 0.8% were BRANCHED-CHAIN-AA-SYN-PWY, PWY-7229, PWY-5686, PWY-3001, ANAGLYCOLYSIS-PWY, PWY-6126, THRESYN-PWY, PWY-2942, PWY-7111, PWY-5667, and PWY0-1319.

**Figure 6:**
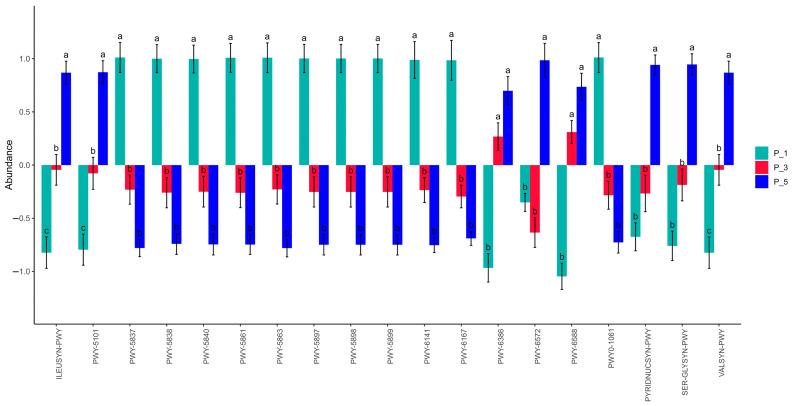
The significantly altered MetaCyc pathways of the fecal microbial community among lactating Bactrian camels. The colors green, red, and blue correspond to P_1 (10 female lactating camels, 1st parity), P_3 (10 female lactating camels, 3rd parity), and P_5 (10 female lactating camels, 5th parity), respectively. The horizontal axis denotes the MetaCyc pathways that display significant changes, each differentiated by color. The vertical axis represents the values for the MetaCyc pathways. If the letters on the bars between two groups differ, they denote significant disparity (*p* < 0.05); otherwise, the difference is considered insignificant.

As depicted in Figure 6, the relative abundances of PWY-5837 (*p* = 0.0003), PWY-5840 (*p* = 0.0003), PWY-5838 (*p* = 0.0003), PWY-5863 (*p* = 0.0003), PWY-5861 (*p* = 0.0004), PWY-5897 (*p* = 0.0004), PWY-5898 (*p* = 0.0004), PWY-5899 (*p* = 0.0004), PWY-6141 (*p* = 0.0002), PWY-6167 (*p* = 0.0002), and PWY0-1061 (*p* = 0.0007) were significantly greater in the P_1 group than in the other groups. On the other hand, the relative abundances of VALSYN-PWY (*p* = 0.0013), SER-GLYSYN-PWY (*p* = 0.0004), PYRIDNUCSYN-PWY (*p* = 0.0006), PWY-6572 (*p* = 0.0014), PWY-5104 (*p* = 0.0014), and ILEUSYN-PWY (*p* = 0.0013) were significantly greater in the P_5 group than in the other groups.

**Figure 7:**
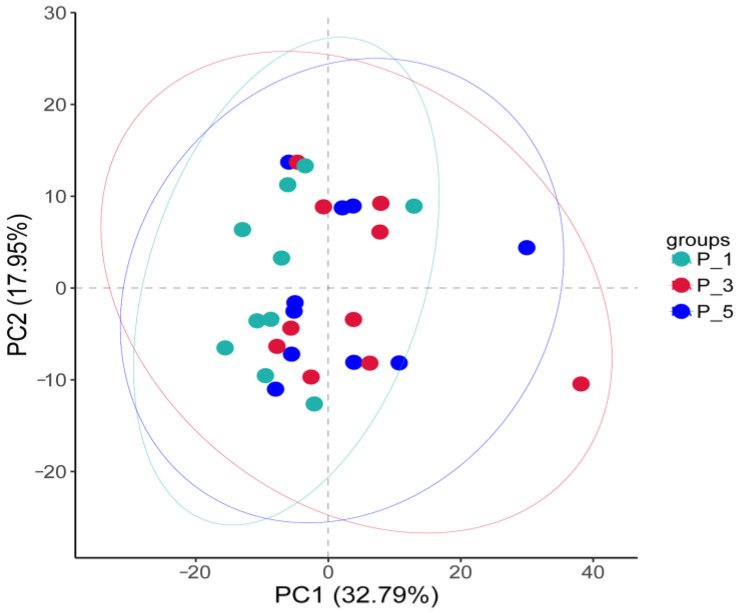
Principal component analysis (PCA) of the MetaCyc pathways in the feces of lactating Bactrian camels. The colors green, red, and blue represent P_1 (10 female lactating camels, 1st parity), P_3 (10 female lactating camels, 3rd parity), and P_5 (10 female lactating camels, 5th parity), respectively. Each plot corresponds to a unique sample, and plots bearing the same color are representative of identical groups.

However, Figure 7 shows that most of the samples clustered within the circle representing the P_1 group, with only two samples scattered outside. PCA 1 accounted for 32.79% of the variation, and PCA 2 accounted for 17.95% of the variation.

### 3.4. Dynamics of the KEGG Pathways in the Lactating Camel Feces

**Figure 8:**
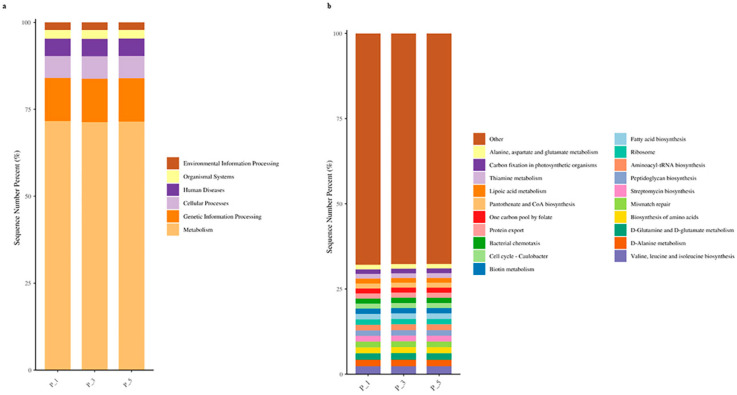
Bar graphs describing KEGG pathways at level 1 (**a**) and level 3 (**b**) in lactating Bactrian camel feces. The horizontal axis represents parities P_1 (10 female lactating camels, 1st parity), P_3 (10 female lactating camels, 3rd parity), and P_5 (10 female lactating camels, 5th parity). The vertical axis, or sequence number percentage, visualizes the percentage of sequences attributed to the corresponding Kyoto Encyclopedia of Genes and Genomes (KEGG) pathway. The color scheme for the bar graphs, moving from top to bottom, corresponds to the colors for each KEGG pathway displayed on the right, ranging from the lowest to the highest relative abundances, with ‘Other’ being an exception. At level 3, ‘Other’ signifies the cumulative percentage of all other KEGG pathways that did not constitute the top 20.

As depicted in Figure 8a, at KEGG level 1, the relative abundance of metabolic pathways was highest, accounting for 71.44% of the total. Genetic information processing accounted for 12.46%, cellular processes accounted for 6.40%, human diseases accounted for 5.00%, organismal systems accounted for 2.53%, and environmental information processing accounted for 2.16%. At KEGG level 3, there were a total of 365 pathways. Figure 8b displays the top 20 pathways, with valine, leucine, and isoleucine biosynthesis being the most abundant at 2.34%, followed by D-alanine metabolism at 1.90%, D-glutamine and D-glutamate metabolism at 1.90%, biosynthesis of amino acids at 1.75%, and mismatch repair at 1.72%.

**Figure 9:**
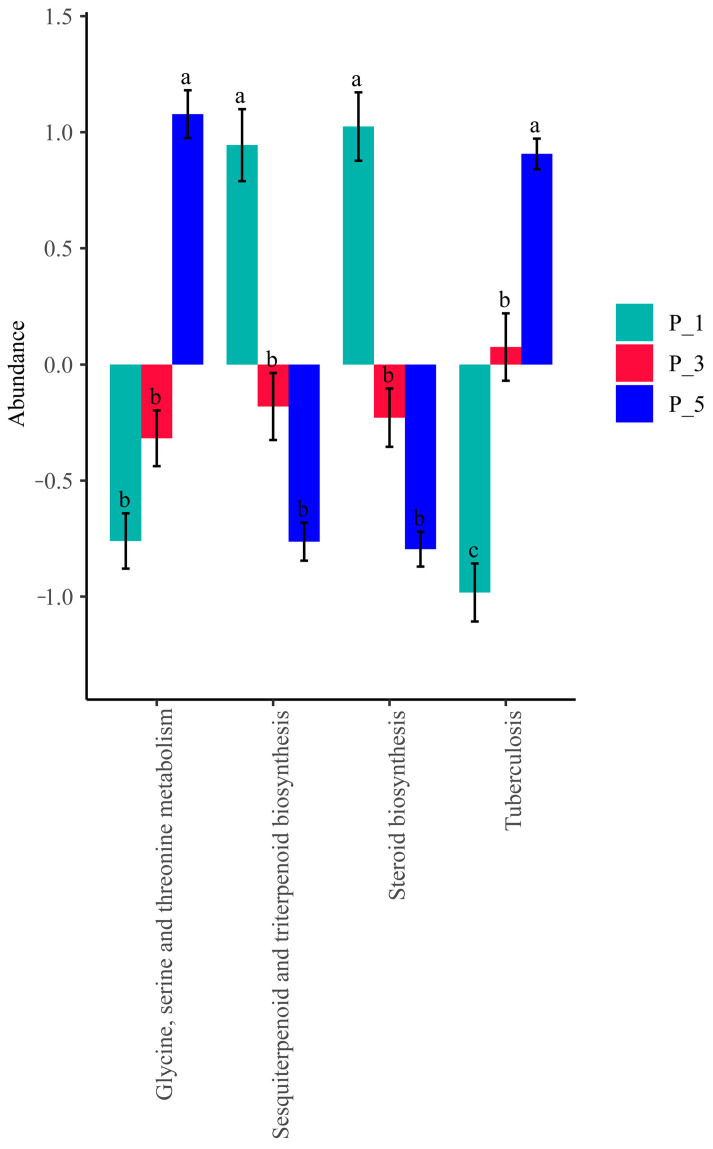
Significantly enriched KEGG pathways associated with the fecal microbial community among lactating Bactrian camels. The colors green, red, and blue correspond to P_1 (10 female lactating camels, 1st parity), P_3 (10 female lactating camels, 3rd parity), and P_5 (10 female lactating camels, 5th parity), respectively. The horizontal axis denotes the KEGG pathways that display significant changes, each differentiated by color. The vertical axis represents the values for the KEGG pathways. If the letters on the bars between two groups differ, they denote significant disparity (*p* < 0.05); otherwise, the difference is considered insignificant.

Figure 9 shows that the relative abundance of the steroid biosynthesis pathway was significantly greater in the P_1 group than in the other groups (*p* = 0.0014). Similarly, the pathway of sesquiterpenoid and triterpenoid biosynthesis was more abundant in the P_1 group (*p* = 0.016).

On the other hand, the relative abundance of the glycine, serine, and threonine metabolism pathways was significantly greater in the P_5 group than in the other groups (*p* = 0.0004), as was the tuberculosis pathway (*p* = 0.00072) in feces.

**Figure 10:**
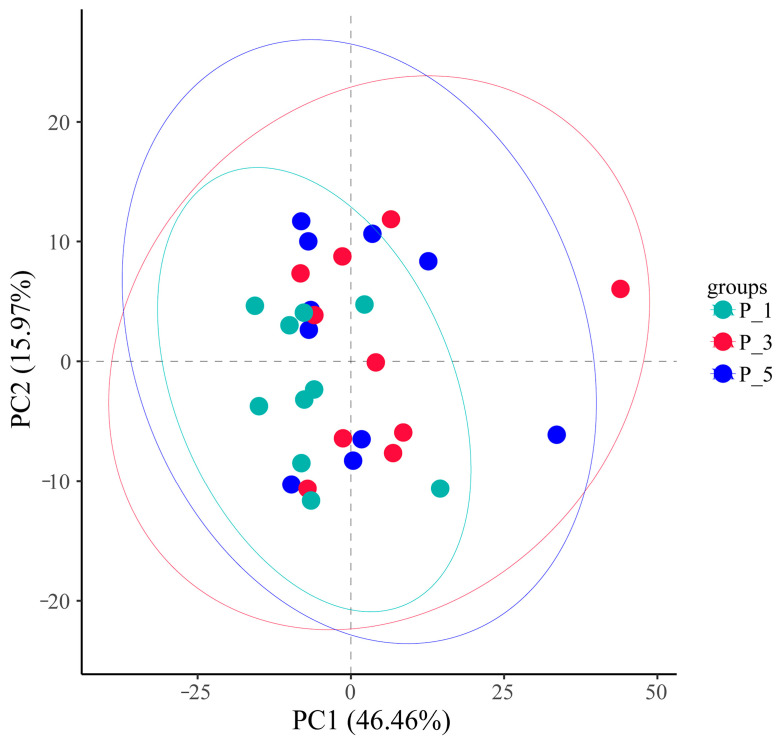
Principal component analysis (PCA) of the KEGG pathways in the feces of lactating Bactrian camels. The colors green, red, and blue represent P_1 (10 female lactating camels, 1st parity), P_3 (10 female lactating camels, 3rd parity), and P_5 (10 female lactating camels, 5th parity), respectively. Each plot corresponds to a unique sample, and plots bearing the same color are representative of identical groups.

However, Figure 10 shows that most of the samples clustered within the circle representing the P_1 group, with only four samples scattered outside. The horizontal axis represents PC1 (principal component 1), which explains 46.46% of the differences among samples, whereas the vertical axis represents PC2 (principal component 2), which contributes 15.97% of the differences among samples.

### 3.5. Dynamics of ECs in Lactating Camel Feces

**Figure 11:**
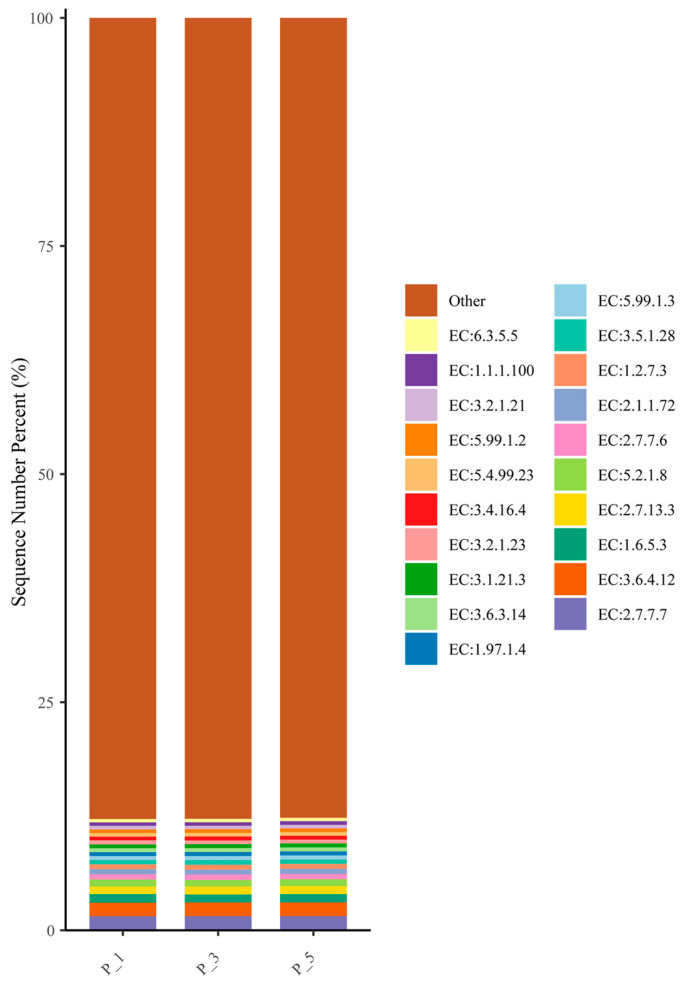
Bar graphs showing the top 20 relatively abundant ECs in lactating Bactrian camel feces. The horizontal axis denotes the parities P_1 (10 female lactating camels, 1st parity), P_3 (10 female lactating camels, 3rd parity), and P_5 (10 female lactating camels, 5th parity). The vertical axis, referred to as the sequence number percentage, illustrates the percentage of sequences allocated to the associated Enzyme Commission numbers (ECs). The colors of the bar graphs, arranged from top to bottom, correspond to the colors of each EC on the right, organized from the lowest to the highest relative abundances, with ‘Other’ being an exception. ‘Other’ represents the aggregate percentage of other KEGG pathways that did not make the top 20.

As depicted in Figure 11, the top 20 relatively abundant ECs were as follows: EC:2.7.7.7 (DNA-directed DNA polymerase) 1.54%, EC:3.6.4.12 (DNA helicase) 1.51%, EC:1.6.5.3 (NADH:ubiquinone reductase (H(+)-translocating)) 0.90%, EC:2.7.13.3 (histidine kinase) 0.87%, and EC:5.2.1.8 (peptidylprolyl isomerase) 0.75%.

**Figure 12:**
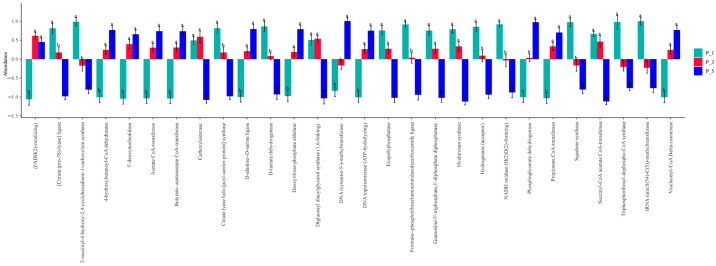
ECs of the fecal microbial community significantly changed among the lactating Bactrian camels in the different groups. In this depiction, green symbolizes P_1 (10 female lactating camels in their 1st parity), red stands for P_3 (10 female lactating camels in their 3rd parity), and blue signifies P_5 (10 female lactating camels in their 5th parity). The horizontal axis denotes the ECs that exhibit substantial changes, whereas the vertical axis signifies their values for ECs. If the lettering on the bar between two groups differs, it indicates a significant variation (*p* < 0.05); if not, the difference is considered statistically insignificant.

As shown in Figure 12, the relative abundances of [citrate (pro-3S)-lyase] ligase (EC:6.2.1.22), 2-succinyl-6-hydroxy-2,4-cyclohexadiene-1-carboxylate synthase (EC:4.2.99.20), citrate lyase holo-[acyl-carrier protein] synthase (EC:2.7.7.61), D-lactate dehydrogenase (EC:1.1.28), formate-phosphoribosylaminoimidazolecarboxamide ligase (EC:6.3.4.23), hydrogenase (acceptor) (EC:1.12.99.6), and NADH oxidase (H2O2-forming) (EC:1.6.3.3) were significantly greater in the feces of P_1 than in those of the other groups. The relative abundances of DNA (cytosine-5-)-methyltransferase (EC:2.1.1.37) and phosphoglycerate dehydrogenase (EC:1.1.95) were significantly greater in the feces of P_5 than in those of the other groups. There were 10 kinds of ECs in the P_3 or P_5 group that were greater than those in the P_1 group, whereas 5 kinds of ECs in the P_3 or P_1 group were greater than those in the P_5 group.

**Figure 13:**
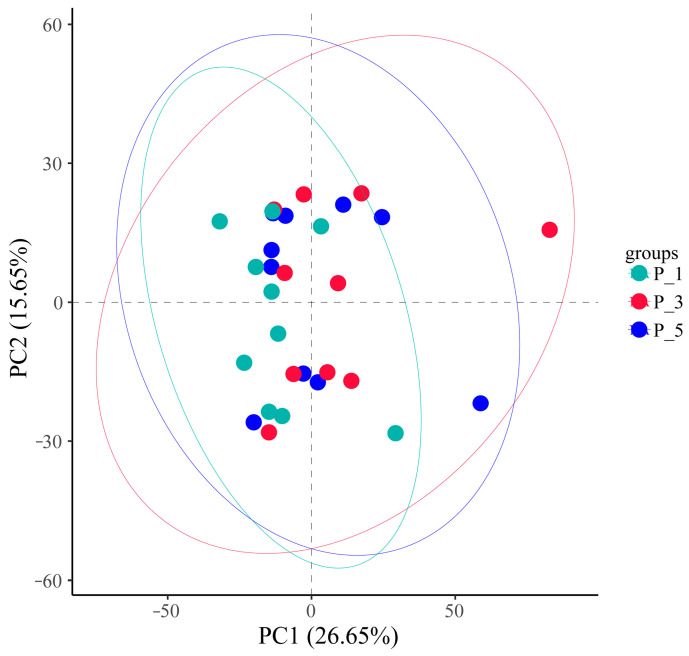
Principal component analysis (PCA) of ECs in the feces of lactating Bactrian camels. In this representation, brown denotes P_1 (10 female lactating camels, first parity), Red corresponds to P_3 (10 female lactating camels, third parity), and Green signifies P_5 (10 female lactating camels, fifth parity). Each plotted point denotes a single sample, and points with identical colors are part of the same group.

However, Figure 13 shows that most of the samples clustered within the circle representing the P_1 group, with the exception of four samples. The horizontal axis represents PC1 (principal component 1), which contributes 26.65% of the differences among samples, whereas the vertical axis represents PC2 (principal component 2), which contributes 15.65% of the differences among samples.

### 3.6. Correlation Between the Top 20 Genera and Antioxidants in Milk and Serum

**Figure 14:**
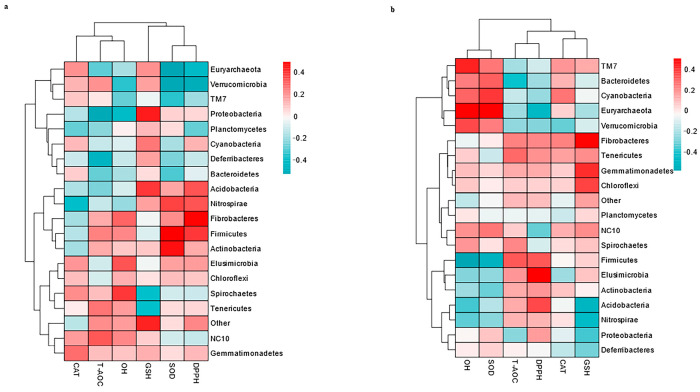
Spearman’s correlation between the top 20 genera associated with the antioxidants in milk (**a**) and the antioxidants in serum (**b**) in lactating Bactrian camels. In the vertical direction, the figures represent the values for the top 20 genera in the feces, whereas the horizontal direction represents the values for the antioxidant parameters. Positive correlation coefficients are depicted in red, with darker shades indicating stronger positive correlations. Negative correlation coefficients are represented in green, with deeper shades indicating stronger negative correlations. The bar on the right displays the correlation coefficient values (r).

As depicted in Figure 14, no genera significantly correlated with the antioxidants in milk and serum.

### 3.7. Correlations Between the Top 20 MetaCyc and Antioxidants in Milk and in Serum

**Figure 15:**
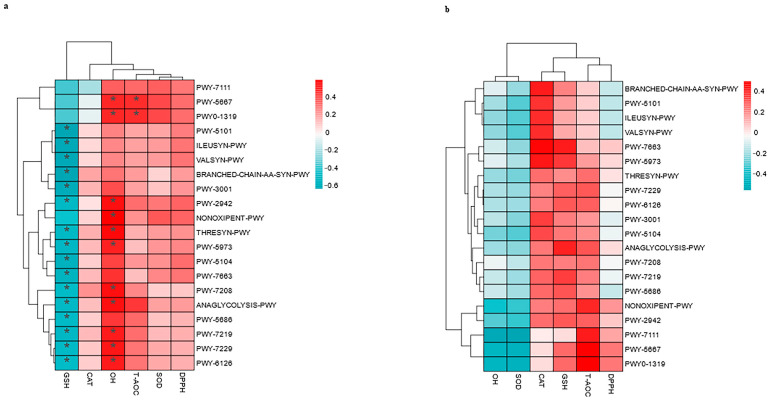
Spearman’s correlation between the fecal top 20 MetaCyc and the antioxidants in milk (**a**) and the antioxidants in serum (**b**) in lactating Bactrian camels. In the vertical direction, the figures represent the values for the top 20 MetaCyc in the feces, whereas the horizontal direction represents the values for the antioxidant parameters. Significant correlations (*p* < 0.05) are denoted by *. Positive correlation coefficients are depicted in red, with darker shades indicating stronger positive correlations. Negative correlation coefficients are rep-resented in green, with deeper shades indicating stronger negative correlations. The bar on the right displays the correlation coefficient values (r).

As depicted in Figure 15b, there was no significant correlation with the antioxidants in the serum. As depicted in Figure 15a, OH was significantly correlated with NONOXIPENT-PWY (pentose phosphate pathway (PPP), r = 0.5894, *p* = 0.0171) and ANAGLYCOLYSIS-PWY (r = 0.5247, *p* = 0.0339). However, ANALYCOLYSIS-PWY (r = −0.4870, *p* = 0.0374) was negatively correlated with GSH, as displayed in Figure 15b. The dual role of ANALYCOLYSIS-PWY was positively linked to OH but negatively related to GSH, which may highlight a trade-off between metabolic output and redox balance.

In addition, the OH in milk is related to PWY-7208 (the superpathway of pyrimidine nucleobase salvage, r = 0.5249, *p* = 0.0339), PWY-7219 (adenosine ribonucleotides de novo biosynthesis, r = 0.4995, *p* = 0.0349), PWY-7229 (adenosine deoxyribonucleotides de novo biosynthesis I, r = 0.5038, *p* = 0.0349), and PWY-6126 (adenosine nucleotides de novo biosynthesis, r = 0.4991, *p* = 0.0349). Nevertheless, PWY-7208 (r = −0.4557, *p* = 0.0431), PWY-7219 (r = −0.4991, *p* = 0.0349), PWY-7229 (r = −0.4748, *p* = 0.0374), and PWY-6126 (r = −0.4619, *p* = 0.0407) were negatively correlated with GSH. The negative correlation of these compounds with GSH may reflect resource competition for nucleotide synthesis against OH.

Moreover, OH was positively correlated with PWY-2942 (L-lysine biosynthesis III, r = 0.5178, *p* = 0.0349), THRESYN-PWY (superpathway of L-threonine biosynthesis, r = 0.5321, *p* = 0.0339), and PWY-5973 (cis-vaccenate biosynthesis, r = 0.4808, *p* = 0.0374). However, GSH was negatively correlated with PWY-5973 (r = −0.4939, *p* = 0.0369), PWY-2942 (r = −0.4766, *p* = 0.0374), and THRESYN-PWY (r = −0.4817, *p* = 0.0374). Pathways such as L-lysine biosynthesis and L-threonine biosynthesis are likely involved in amino acid synthesis. The negative correlation of these genes with GSH may reflect resource competition between amino acid synthesis and glutathione regeneration.

However, as depicted in Figure 15a, GSH was negatively correlated with PWY-5101 (L-isoleucine biosynthesis II, r = −0.6350, *p* = 0.0131), ILEUSYN-PWY (L-isoleucine biosynthesis I, r = −0.6072, *p* = 0.0131), VALSYN-PWY (r = −0.6072, *p* = 0.0131), branched-chain-AA-SYN-PWY (r = −0.6232, *p* = 0.0131), PWY-3001 (superpathway of L-isoleucine biosynthesis I, r = −0.5803, *p* = 0.0181), PWY-7663 (gondoate biosynthesis, r = −0.5269, *p* = 0.0339), and PWY-5104 (L-isoleucine biosynthesis IV, r = −0.5451, *p* = 0.0339).

In addition, PWY-5667 (CDP-diacylglycerol biosynthesis I) and PWY0-1319 (CDP-diacylglycerol biosynthesis II) were positively correlated with both T-AOC (r = 0.4608, *p* = 0.0407; r = 0.4776, *p* = 0.0374) and OH (r = 0.4608, *p* = 0.0407; r = 0.4776, *p* = 0.0374), respectively.

### 3.8. Correlation Between Top 20 KEGG Pathways and the Antioxidants in Milk and in Serum

**Figure 16:**
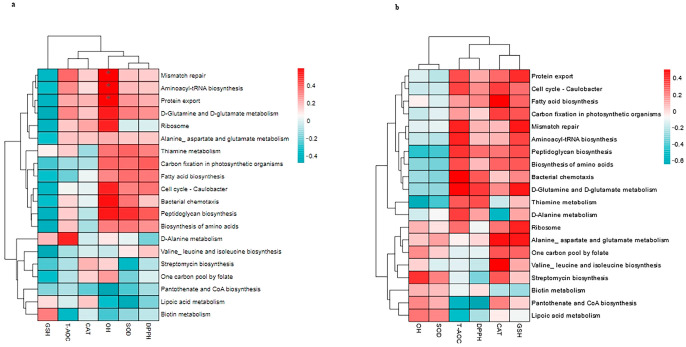
Spearman’ correlation between the fecal top 20 KEGG pathways and the antioxidant in milk (**a**), and the antioxidant in serum (**b**) in lactating Bactrian camel. In the vertical direction, the figures represent the values for the fecal top 20 KEGG pathways, while the horizontal direction represents the values for the antioxidant parameters. Significant correlations (*p* < 0.05) are denoted by *. Positive correlation coefficients are depicted in red, with darker shades indicating stronger positive correlations. Negative correlation coefficients are represented in green, with deeper shades indicating stronger negative correlations. The bar on the right displays the correlation coefficient values (r).

As depicted at Figure 16a, OH positively correlated with mismatch repair (r = 0.5961, *p* = 0.0422), aminoacyl-tRNA biosynthesis (r = 0.5898, *p* = 0.0422), and protein repair (r = 0.5651, *p* = 0.04); whereas there was no significant correlation displayed at Figure 16b. Furthermore, T-AOC, SOD, CAT, and DPPH in milk also showed no significant correlation with the 20 KEGG pathways. In milk, OH’s association with specific pathways suggests compartmentalized antioxidant responses, distinct from other antioxidant parameters.

### 3.9. Correlation Between Top 20 ECs and the Antioxidants in Milk and in Serum

**Figure 17:**
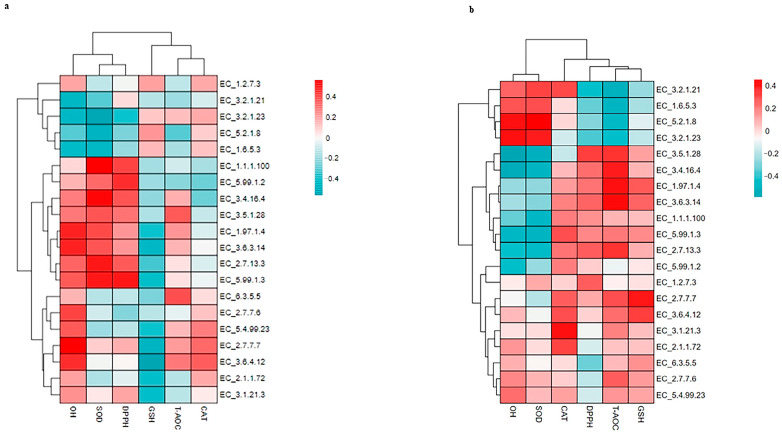
Spearman’s correlation between the fecal Top 20 ECs and the antioxidants in milk (**a**) and the antioxidants in serum (**b**) in lactating Bactrian camels. In the vertical direction, the figures represent the values for the top 20 ECs in the feces, whereas the horizontal direction represents the values for the antioxidant parameters. Positive correlation coefficients are depicted in red, with darker shades indicating stronger positive correlations. Negative correlation coefficients are represented in green, with deeper shades indicating stronger negative correlations. The bar on the right displays the correlation coefficient values (r).

As depicted in Figure 17, no significant correlation was detected between the top 20 ECs and the antioxidants in milk. In addition, no EC was significantly correlated with the antioxidants in the serum. This is a limitation of this study, and further studies involving a larger population are needed to evaluate these results.

## 4. Discussion

The Camelidae family comprises the Bactrian camel (*Camelus bactrianus*), the dromedary camel (*Camelus dromedarius*), and four species of South American camelids: llama (*Lama glama*), alpaca (*Lama pacos*) guanaco (*Lama guanicoe*), and vicuña (*Vicugna vicugna*) [31]. Camels hold significant cultural significance and have been a vital contributor to arid-land ecologically sustainable development [32], while there has been an increase in the breeding and management of camels for the purpose of milk production [33].

In this study, prior to the first parturition, the camels remained in the traditional rangeland production system, where the main vegetation belongs to Amaranthaceae, Fabaceae, Zygophyllaceae, Poaceae, and Liliaceae. Owing to the palatability of Mongolian rangeland plants, camels can consume 93 plant species year round [34]. In summer, camels mainly graze on herbs, whereas in winter, camels prefer to browse shrubs [35]. After being returned to the extensive system, the camels were provided with the same diet across the different parities.

Camel milk is the key component of the human diet, especially in arid and semi-arid regions, highlighting its nutritional importance, pharmaceutical potential, and medicinal value [36,37]. In addition, camel milk has been found to have antioxidant, anti-inflammatory, and immunomodulatory effects [38,39]. In dromedaries, the malondialdehyde (MDA) level in milk much greater than the MDA level in serum [4]. In the serum, the GSH (mean level of 31.23 mg/dL) during the lactating period higher than the GSH level in pregnant dromedary camels [14]. In Bactrian camels, the average T-AOC (mean level of 28.40 mg/mL) in milk decreases as parity increases in another report [40]. Its antioxidant parameters, such as the T-AOC, were much greater than the corresponding serum parameters in the present study; the T-AOC was 59.74 mg/mL (SEM = 4.1194) in the milk of the primiparous camel and 4.82 mg/mL (SEM = 0.0534) in the serum of the primiparous camel.

Compared with multiparous camels, primiparous camels had lower milk production rates and percentages of bimodal curves [41]. At the fifth parity of camels, the highest levels of protein and lactose are reported in camel milk [42]. Parity influences serum antioxidant [43] and milk antioxidant levels [44]. The number of pregnancies significantly reduces the serum levels of some antioxidants [45]. Among dairy cattle, primiparous cows are more susceptible to oxidant antioxidant imbalance than multiparous postpartum cows are [46]. Multiparous cows also present increased antioxidant transcription factor and expression levels in milk [47]. In goats, higher parity is associated with higher levels of antioxidants in serum [48]. In the present study, the amount of OH and SOD in the serum of first-parity lactating camels was significantly greater than that in the other groups, whereas no antioxidant parameters were significantly greater than those in the other groups. Similarly, the SOD concentration decreases with increasing parity in dairy cattle [49].

In both the fecal and rumen microbial communities of adult Bactrian camels, the dominant phyla are Firmicutes, Verrucomicrobia, and Bacteroidetes [50,51]. Another study demonstrated that the fecal microbiota of Bactrian camels at 1 and 3 years of age dominated by the same phyla [52]. In the present study, these three phyla were also found to be most abundant in Bactrian camel feces. The unique gastrointestinal microbiome of Bactrian camels may be due to their distinct digestive systems, and the fecal microbial communities of both domestic and wild Bactrian camels may cluster together [53]. In gastrointestinal tract samples from yaks, Firmicutes, Bacteroidetes, and Verrucomicrobia were the most abundant phyla [54]. Similarly, in beef cattle, the most common phyla in the fecal microbiota include Firmicutes, Bacteroidetes, and Verrucomicrobia [55,56]. However, the dromedary camel fecal microbes and their enzymes are primarily affiliated with Bacteroidetes, Firmicutes, and Proteobacteria [57,58]. Furthermore, the sheep fecal core microbiome under various feeding systems is dominated by Firmicutes, Bacteroidetes, and Proteobacteria [59,60]. In the feces of dairy cattle, the most abundant phyla are Firmicutes, Proteobacteria, and Actinobacteria [61].

In the present study, the overall structure of the fecal microbial communities (Figure 4) and their functions (Figure 7, Figure 10, and Figure 13) in the P_1, P_3, and P_5 samples were clustered together. In dairy cattle, the ruminal bacterial communities exhibit similar diversity between the first and second lactation cycles [62]. However, in first-lactation dairy cows, Bacteroidetes contribute to the majority of metabolic functions, whereas Firmicutes and Proteobacteria increase in abundance during the second and third lactation cycles [63].

In this study, primiparous camels were enriched in Verrucomicrobiaceae and *Akkermansia* and involved in steroid biosynthesis. Previous reports suggest that bacterial species possessing critical genes for steroid biosynthesis belong primarily to the phyla Actinobacteria, Deltaproteobacteria, Gammaproteobacteria, and Verrucomicrobia [64]. Another study classified *Akkermansia muciniphila* as a steroid-producing bacterium that colonizes the mucus layer of the gastrointestinal tract, representing 1 to 4% of the fecal microbiota [65]. This bacterium is known to stimulate mucosal microbial networks and enhance intestinal barrier function, thereby providing essential host immune responses [66].

Furthermore, the abundance of *Clostridium* was highest in third parity lactating camels in this study, whereas the abundance of *Ruminococcus* was significantly greater in fifth parity camels than in other lower parity lactating camels. Both the genera *Clostridium* and *Ruminococcus* belong to Firmicutes, which suggests that camels rich in lignocellulolytic enzymes could be indicative of a fecal microbial community in multiparous camels that is rich in fiber-degrading microbes [67,68]. Finally, this study revealed that the fecal tuberculosis pathway increased with increasing parity, warranting future validation. *Mycobacterium tuberculosis* is reported as the etiological agent of tuberculosis [69,70].

Interestingly, OH in camel milk was associated with both the NONOXIPENT-PWY and ANAGLYCOLYSIS-PWY metabolic pathways in this study. NONOXIPENT-PWY and the glycolysis pathway of anaerobes (ANAGLYCOLYSIS-PWY) were highly enriched in wild-living macaques compared with housed ones [71]. In the present study, the relative abundance of NONOXIPENT-PWY (1.01%) was the highest, whereas that of ANAGLYCOLYSIS-PWY was 0.8%. The NONOXIPENT-PWY pathway is the nonoxidative subpathway of the PENTOSE-Phosphate-PWY (PPP), the common glycolysis pathway for the catabolism of glucose, and it regulates Treg cell function, maintaining immune homeostasis and thereby controlling oxidative phosphorylation [72]. Glycolysis was first studied as a pathway for the utilization of glucose, and ANAGLYCOLYSIS-PWY is also described as glycolysis III [73]. The relative abundance of glycolysis and gluconeogenesis in the rumen contents ranges from 4% to 6% in buffalo [74]. Within the category carbohydrate metabolism, glycolysis/gluconeogenesis and PPP were enriched in the rumens of multiparous mid-lactation Holstein dairy cows [75].

In addition, PWY-5667 and PWY0-1319 were positively correlated with both T-AOC values in milk. These pathways describe the biosynthesis of CDP-diacylglycerol in some gram-negative bacteria [76]. Mitochondria are capable of synthesizing several lipids autonomously, such as phosphatidylglycerol, cardiolipin and, in part, phosphatidylethanolamine, phosphatidic acid, and CDP-diacylglycerol [77]. In addition, the OH in milk is related to PWY-7208, nucleotide metabolism, i.e., the superpathway of pyrimidine nucleobase salvage [78]; PWY-7219, adenosine ribonucleotide de novo biosynthesis [79]; PWY-7229, adenosine deoxyribonucleotide de novo biosynthesis I [80]; and PWY-6126, adenosine nucleotide de novo biosynthesis [81].

However, it is important to note that this study establishes associations between microbes and KEGG pathways or EC numbers using a limited sample size of randomly selected lactating camels at different parities. Importantly, correlation does not necessarily imply causation. Therefore, future investigations should focus on controlled experiments to substantiate these correlations. In addition, this study is limited in terms of the ability of qPCR to add further support because of the limited amount of the sampled materials, as well as the metagenomics and metabolomics analysis.

This study demonstrated the effects of parity on the antioxidant parameters and the fecal microbiome of lactating camels. However, the intensification of camels for milk production necessitates a comprehensive assessment by scientists to determine camel health and welfare, considering the complicated factors affecting milk production [82]. To date, the only tools for evaluating the welfare of camels include a combination of individual, diet and management measures to investigate individual camel health status [83,84]. However, there are still insufficient protocols for assessing the health and welfare of camels [85]. Thus, the level of the oxidative state should be taken into consideration when evaluating the health and welfare of lactating camel herds.

## 5. Conclusions

In the present study, we investigated the influence of parity dynamics on milk antioxidants, serum antioxidants, and the fecal microbial communities of lactating Bactrian camels. The OH, SOD, and DPPHlevels in milk were greater for the fifth parity and third parity groups than for the first parity group. The amount of T-AOC and DPPH was the highest in third-parity camels, in both milk and serum. These differences suggest that parity influences antioxidative resilience, which may affect immune function and camel health. Thus, their antioxidant profiles could inform herd management practices for sustaining welfare in camel breeders.

Furthermore, the amount of hydroxyl radicals in milk was positively associated with the NONOXIPENT-PWY and ANAGLYCOLYSIS-PWY metabolic pathways. In addition, T-AOC was positively associated with CDP-diacylglycerol-related pathways, whereas the OH of camel milk was also related to nucleotide metabolism-related pathways. Primiparous camels were enriched in the Verrucomicrobiaceae and steroid biosynthesis pathways, whereas Clostridiaceae, Bacteroidaceae, Paraprevotellaceae, and Ruminococcaceae were enriched in multiparous camels. Pathways associated with bacterial infection (e.g., KEGG: tuberculosis) were more abundant in multiparous camels, warranting cautious interpretation. Microbial changes might directly or indirectly influence antioxidant capacity, creating a feedback loop between the gut microbiome and oxidative balance. In camels, future research should focus on mechanistic studies to clarify the relationship between the gut microbiota and redox homeostasis in serum and milk.

## Figures and Tables

**Table 1:** Composition and nutrient levels of the diets of lactating Bactrian camels (dry matter basis, %).

Ingredients	Percentages
Corn straw	34.56
Oat grass	12.76
Alfalfa grass	12.32
Whole-plant corn silage	24.9
Concentrate	14.38
NaCl	0.7
Sodium bicarbonate	0.38
CP	9.63
ADF	38
NDF	54.41
Non-fiber carbohydrate	24.09
Starch	10.73
EE	2.27
TDN	56
Ca	0.45
P	0.31

The concentrate (flaked maize, bean cake, calcium perphosphate, and premix) was provided by Yinggesu Biotechnology Co., Ltd. (Bayannur, China). CP, crude protein. ADF, acid detergent fiber. NDF, neutral detergent fiber. EE, ether extract. Total digestible nutrients (TDN).

**Table 2:** Comparison of milk production and antioxidant parameters of milk from lactating Bactrian camels with different parities (*n* = 10).

Parameters	P_1	P_3	P_5	SEM	*p* Value
T-AOC (mg/mL)	59.74 ^b^	86.09 ^a^	56.60 ^b^	4.1194	0.0051
CAT (U/mL)	2.87 ^a^	2.17 ^b^	2.09 ^b^	0.1363	0.0246
OH (U/mL)	10.27 ^b^	16.75 ^a^	16.08 ^a^	0.7475	<0.0001
SOD (U/mL)	43.62 ^b^	68.17 ^a^	64.49 ^a^	3.6173	0.0084
GSH (U/mL)	90.05	76.88	85.24	5.9390	0.6726
DPPH (μg Trolox/mL)	192.28 ^b^	205.43 ^a^	203.25 ^a^	1.8610	0.0071

P_1 (10 female lactating camels, 1st parity); P_3 (10 female lactating camels, 3rd parity), and P_5 (10 female lactating camels, 5th parity). a, b Within a row, means with different superscripts differ significantly (*p* < 0.05).

**Table 3:** Comparison of the antioxidant parameters of the serum of lactating Bactrian camels with different parities (*n* = 10).

Parameters	P_1	P_3	P_5	SEM	*p* Value
T-AOC (mg/mL)	4.82 ^b^	5.30 ^a^	4.93 ^b^	0.0535	<0.0001
CAT (U/mL)	6.63	6.15	7.64	0.2600	0.0609
OH (U/mL)	9.62 ^a^	6.02 ^b^	6.50 ^b^	0.4950	0.0017
SOD (U/mL)	13.64 ^a^	7.97 ^c^	11.50 ^b^	0.5144	<0.0001
GSH (U/mL)	26.05	24.49	24.58	0.6804	0.6000
DPPH (μg Trolox/mL)	116.59 ^b^	126.04 ^a^	121.05 ^b^	16.109	0.0019

P_1 (10 female lactating camels, 1st parity); P_3 (10 female lactating camels, 3rd parity), and P_5 (10 female lactating camels, 5th parity). a, b, c Within a row, means with different superscripts differ significantly (*p* < 0.05).

**Table 4:** Comparison of alpha diversity indices among groups of the fecal microbial community in lactating Bactrian camels with different parities (*n* = 10).

Variable	Group 1	Group 2	*p* Value
faith_pd *	P_5	P_3	1.000
	P_5	P_1	0.796
	P_3	P_1	0.853
Observed OTUs	P_5	P_3	0.143
	P_5	P_1	0.089
	P_3	P_1	0.247
shannon_entropy	P_5	P_3	0.393
	P_5 ^a^	P_1 ^b^	0.015
	P_3 ^a^	P_1 ^b^	0.015
Simpson	P_5	P_3	0.481
	P_5 ^a^	P_1 ^b^	0.023
	P_3	P_1	0.089

* faith_pd represents Faith’s phylogenetic diversity index. P_1 (10 female lactating camels, 1st parity); P_3 (10 female lactating camels, 3rd parity), and P_5 (10 female lactating camels, 5th parity). a, b Within a row, means with different superscripts differ significantly (*p* < 0.05).

## Data Availability

All the sequences were deposited to the NCBI sequence read archive (SRA) at the accession number: PRJNA964482.

## Abbreviations

The following abbreviations are used in this manuscript:

T-AOC	Total antioxidant capacity
CAT	Catalase
OH	Antioxidant activity for hydroxyl radical
SOD	Superoxide dismutase
GSH	Glutathione peroxidase
DPPH	Antioxidant activity for polypeptides
